# Involvement of dopamine D_2_ receptor in the diurnal changes of tuberoinfundibular dopaminergic neuron activity and prolactin secretion in female rats

**DOI:** 10.1186/1423-0127-21-37

**Published:** 2014-05-03

**Authors:** Shu-Ling Liang, Sheng-Chieh Hsu, Jenn-Tser Pan

**Affiliations:** 1 Department of Physiology and Pharmacology, College of Medicine, Chang Gung University, Tao-Yuan 33302, Taiwan; 2 Department of Biomedical Sciences, College of Medicine, Chang Gung University, Tao-Yuan 33302, Taiwan; 3 Department of Biological Sciences, Oakland University, Rochester, MI 48309, USA

**Keywords:** Circadian rhythm, 3, 4-dihydroxyphenylacetic acid (DOPAC), Prolactin, Dorsal medial arcuate nucleus, Median eminence, Dopamine receptor

## Abstract

**Background:**

An endogenous dopaminergic (DA) tone acting on D_3_ receptors has been shown to inhibit tuberoinfundibular (TI) DA neuron activity and stimulate prolactin (PRL) surge in the afternoon of estrogen-primed ovariectomized (OVX+E_2_) rats. Whether D_2_ receptor (D_2_R) is also involved in the regulation of TIDA and PRL rhythms was determined in this study.

**Results:**

Intracerebroventricular (icv) injection of PHNO, a D_2_R agonist, in the morning inhibited TIDA and midbrain DA neurons’ activities, and stimulated PRL secretion. The effects of PHNO were significantly reversed by co-administration of raclopride, a D_2_R antagonist. A single injection of raclopride at 1200 h significantly reversed the lowered TIDA neuron activity and the increased serum PRL level at 1500 h. Dopamine D_2_R mRNA expression in medial basal hypothalamus (MBH) exhibited a diurnal rhythm, i.e., low in the morning and high in the afternoon, which was opposite to that of TIDA neuron activity. The D_2_R rhythm was abolished in OVX+E_2_ rats kept under constant lighting but not in OVX rats with regular lighting exposures. Pretreatment with an antisense oligodeoxynucleotides (AODN, 10 μg/3 μl/day, icv) against D_2_R mRNA for 2 days significantly reduced D_2_R mRNAs in central DA neurons, and reversed both lowered TIDA neuron activity and increased serum PRL level in the afternoon on day 3. A diurnal rhythm of D_2_R mRNA expression was also observed in midbrain DA neurons and the rhythm was significantly knocked down by the AODN pretreatment.

**Conclusions:**

We conclude that a diurnal change of D_2_R mRNA expression in MBH may underlie the diurnal rhythms of TIDA neuron activity and PRL secretion in OVX+E_2_ rats.

## Background

Dopamine (DA) is a well-established neurohormone that is synthesized by tuberoinfundibular dopaminergic (TIDA) neurons in hypothalamic dorsal medial arcuate nucleus (dmARN) and released from the median eminence (ME). It is then delivered to anterior pituitary by hypothalamo-hypophysial portal vessels and acts as a prolactin (PRL)-inhibiting hormone [1,2].

Intracerebroventricular (icv) injection of DA inhibits basal TIDA neuron activity in estrogen-primed ovariectomized (OVX+E_2_) rats [3]. Single-unit recording of dmARN neurons in brain slices obtained from OVX+E_2_ rats reveals that DA inhibits the firing rates of 63-74% dmARN neurons recorded [4-7], and the inhibitory effect is mimicked by selective D_2_ and D_3_ receptor (D_2_R and D_3_R) agonists [5]. The percentage of DA-inhibited dmARN neurons is significantly reduced (to 39-44%) in brain slices obtained from OVX+E_2_ rats pretreated with antisense oligodeoxynucleotides (AODN) against D_2_R or D_3_R mRNA for 2 days [6]. Combined treatment of both AODNs reduces the percentage even further (to 18%). Moreover, the firing rates of the DA-inhibited dmARN neurons exhibit a diurnal change similar to the rhythm observed in TIDA neuron activity as determined by neurochemical methods [7]. All the above findings indicate that TIDA neurons possess D_2_-like autoreceptors that may mediate the feedback control of their own activities.

The diurnal rhythm of TIDA neurons, i.e., high in the morning and low in the afternoon, is essential for the estrogen-induced PRL afternoon surge [8,9]. We recently reported that DA acting on D_3_R inhibits basal TIDA neuron activity, and an endogenous DA tone acting on D_3_R may be involved in the diurnal changes of TIDA neuron activities and PRL secretion in OVX+E_2_ rats [10]. Whether D_2_R is involved was determined in this study.

It has been shown that both mouse striatal D_3_R mRNA level [11] and D_2_/D_3_R proteins [12] exhibit diurnal rhythms, which may correlate with motor functions. There is no report, however, on D_2_R mRNA level in medial basal hypothalamus (MBH) where TIDA neurons reside. Thus, we determined the diurnal changes of MBH D_2_R mRNA expression in this study. The D_2_R mRNAs in midbrain DA neurons were also measured for comparison.

PHNO and raclopride, selective D_2_R agonist and antagonist respectively, and AODN against D_2_R mRNA were used in this study. TIDA neuron activity was determined by measuring DA and its metabolite DOPAC in the ME, where TIDA neurons terminate. For comparison, the activities of nigrostriatal (NS) and mesolimbic (ML) DA neurons were also determined. The amounts of D_2_R mRNA in MBH, substantia nigra (SN) and ventral tegmental area (VTA) were determined by quantitative reverse transcription-polymerase chain reaction (QRT-PCR). The results clearly show that D_2_R may play a significant role in regulating diurnal rhythms of TIDA neuron activity and PRL secretion.

## Methods

### Animal sources and treatments

Adult female Sprague–Dawley rats (6- to 8-week-old) weighing between 220 and 250 g were purchased from National Yang-Ming University Animal Center (Taipei, Taiwan, ROC) and BioLASCO Taiwan Co., Ltd (Taipei, Taiwan, ROC). All animals were housed in a light (lights on between 06:00 and 20:00 h)- and temperature (23 ± 1°C)-controlled room with free access to tap water and rat chow except otherwise notice. All rats used in the experiments were surgically OVX one week before they were implanted subcutaneously each with a capsule (silicone tubing, A-M systems, Everett, WA, USA; id, 1.57 mm; od, 3.18 mm; active length, 20 mm) containing 17β-estradiol (E_2_; 150 μg/ml corn oil; Sigma-Aldrich, St. Louis, MO, USA) except otherwise notice. Plasma E_2_ levels of implanted animals are equivalent to proestrous levels as previously reported [13]. Each rat also received implantation of a single icv cannula (23-gauge stainless steel) in the right lateral cerebroventricle using a stereotaxic instrument (DKI 900; David Kopf Instruments, Tujunga, CA, USA) at the same time when the E_2_ capsule was implanted. Ether or isoflurane were used as anesthetics for ovariectomy, and equithesin (2 ml/kg BW, ip) or sodium pentobarbital (50 mg/kg BW, ip) were used in stereotaxic surgery. All experiments were performed one week after the implantations.

For icv injections of specific D_2_ agonist, antagonist and AODN, a 30-gauge needle connected to a microsyringe (10 μl) with PE-10 tubing was inserted into the pre-implanted cannula in each conscious, free moving rat. All chemicals and vehicles were slowly injected (3 μl/2 min) into the right lateral cerebroventricle and the animals were decapitated at specific times afterwards without anesthesia.

The handlings and surgical procedures of animals were in accordance with the protocols approved by the Institutional Animal Care and Use committee of Chang Gung University.

### Experimental designs and sample collections

In the first study, both time- and dose-dependent effects of PHNO were determined in the morning around 1000 h. Groups of rats were sacrificed at 15, 30, 60 or 120 min following the injection of PHNO (0.1 μg, icv). Rats in the control group received injections of artificial cerebrospinal fluid (aCSF) and were decapitated at specific time points afterwards. They were pooled as one control group since no significant difference was observed. In the dose-dependent study, groups of rats received various doses of PHNO (0.001-0.1 μg, icv) and they were sacrificed at 30 min after the injection. One group of rats received combined injection of PHNO (0.1 μg) and raclopride (0.1 μg), a D_2_R antagonist.

In the second study, a group of rats received injections of raclopride (10 μg/3 μl, icv) around 1200 h on the experimentation day and they were sacrificed around 1500 h on the same day. Rats in the control groups received vehicle injections and were sacrificed either in the morning or afternoon on the same day.

In the third study, the D_2_R mRNA expressions in the morning and afternoon and the responses to constant light and absence of E_2_ exposure were determined. Rats were OVX on day 1 and divided into three groups with different treatments on day 7; the first group of rats was implanted with the E_2_ and raised under regular light–dark cycle (OVX+E_2_, LD), the second group of rats was implanted with the E_2_ and raised under constant light (OVX+E_2_, LL), the third group of rats was without E_2_ implantation and raised under regular light–dark cycle (OVX, LD). All the rats were sacrificed in the morning and afternoon on day 14.

In the fourth study, OVX+E_2_ rats were divided into three groups; each received daily icv injections of aCSF, AODN or random AODN against D_2_R mRNA (10 μg/3 μl) for 2 days. On day 3, each group was further divided into two subgroups: one was sacrificed in the morning and the other in the afternoon.

After sacrifice, the rat’s brain was quickly removed from the skull and frozen on dry ice. Thick (600 μm) coronal brain sections were prepared with a cryostat and thaw mounted onto glass slides. ME, MBH, SN, VTA, dorsal lateral striatum (dlST) and nucleus accumbens (NA) were removed using a modified micropunch technique [14]. Briefly, the tissues were punched using a stainless steel needle from one or two frozen slices that are 2.2-1.0 mm anterior to bregma for punching dlST and NA, 2.0-3.2 mm posterior to bregma for punching ME and MBH, and 4.8-6.0 mm posterior to bregma for punching VTA and SN [15].

For measurement of their monoamine contents, the punched brain tissues of ME, dlST and NA from each rat were individually stored in 40 μl of 0.15 M sodium phosphate buffer containing 0.65 mM sodium octanesulphonate, 0.5 mM EDTA, and 12% methanol, with its pH adjusted to 2.6 using phosphoric acid, and kept at −20°C until assayed by high-performance liquid chromatography (HPLC) plus electrochemical detection. For quantitative analysis of D_2_ mRNA level, the punched brain tissues of MBH, VTA and SN from each rat were placed individually in eppendorf vials and stored at −80°C until assayed by QRT-PCR. The trunk blood of each rat was individually collected right after decapitation, and the serum was obtained after centrifugation of the coagulated blood and stored at −20°C until assayed for its PRL content by radioimmunoassay (RIA).

### QRT-PCR

The punched brain tissues from two to three animals of the same treatment group were pooled together for RNA extractions using the RNeasy lipid tissue kit (QIAGEN, Hilden, Germany). The cDNA was then synthesized from mRNA using the RevertAid™ First Strand cDNA Synthesis kit (ThermoFisher Scientific, Rockford, IL, USA). Quantitative PCR reactions were carried out using the Maxima SYBR Green/ROX qPCR master mix (ThermoFisher Scientific, Rockford, IL, USA) in a PCR machine (7500 Fast Real-Time PCR system, Applied Biosystem, Carlsbad, CA, USA). Relative quantification of D_2_R mRNA was obtained via the comparative CT method, and the relative amounts of the targets were normalized to their own endogenous control GAPDH. The primers used for amplification were as follows: GAPDH (BC059110): (forward) 5′-acagcaacagggtggtggac-3′ and (reverse) 5′-tttgagggtgcagcgaactt-3′; D_2_R (X56065.1): 5′-gtcctctacagcgccttcac-3′ and (reverse) 5′-atgaggtctggcctgcatag- 3′. PCR cycle conditions were as follows: 15 s at 95°C, 60 s at 60°C for 45 cycles. The assays were performed at least twice for all samples.

### Resources of chemicals and preparations

PHNO (a gift from Merck, Sharp and Dohme, Whitehouse Station, NJ, USA) and raclopride (RBI, Natick, MA, USA) were dissolved in distilled water. The D_2_ AODN was a 19-mer (5′-AGG-ACA-GGT-TCA-GTG-GAT-C-3′) complementary to codons 2–8 (nucleotides 4–22) of the D_2_R mRNA. The random sequence for D_2_ AODN (5′-AGA-ACG-GCA-CTT-AGT-GGG-T-3′), was used as control. All sequences were adopted from previous studies [6,16] and were synthesized by a local company (Watson Biotechnology Co. LTD. Taipei, Taiwan) or IDT (Integrated DNA Technologies, Inc. Coralville, IA, USA). The ODN were phosphorothioate-modified to increase the resistance from degradation by endogenous nucleases [17,18], and they were dissolved in aCSF for icv injections. The composition of aCSF is as follows (in mM): NaCl 125.1, KCl 3.8, KH_2_PO_4_ 1.2, MgSO_4_ 1.3, CaCl_2_ 2.4, NaHCO_3_ 26, and dextrose 10.

### Chemical assays and statistical analysis

Dopamine and its metabolite 3,4-dihydroxyphenylacetic acid (DOPAC) levels in the ME, dlST and NA were determined by HPLC with electrochemical detection as previously described [3,8,9,19]. Protein contents in punched brain tissues used for HPLC were measured by the Lowry method [20]. Activities of TIDA, NSDA and MLDA neurons were estimated using the index of DOPAC/DA ratio. Materials used in PRL RIA were kindly provided by Dr. A.F. Parlow of the National Hormone and Pituitary Programme of NIDDK, USA.

Statistical analyses were conducted using either two-way or one-way ANOVA for testing significant differences among time points and/or treatments. One-way ANOVA followed by the Student-Newman-Keuls’ multiple range test were performed for all groups. Differences were considered significant at p < 0.05 or p < 0.01.

## Results

### Effects of PHNO, a D_2_ receptor agonist, on basal TIDA, NSDA and MLDA neuron activities and serum PRL levels in OVX+E_2_ rats

**Figure 1:**
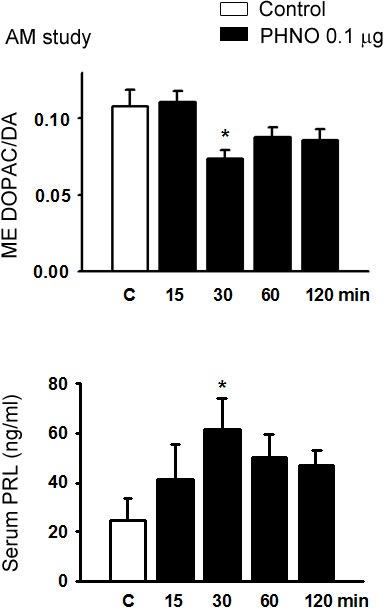
**Effects of a D**_**2 **_**receptor agonist, PHNO (0.1 μg/3 μl, icv), on ME DOPAC/DA ratio (upper) and serum PRL (lower) level in OVX+E**_**2 **_**rats in the morning.** Data are expressed as mean ± SEM (n = 5–7 rats). *, p < 0.05 compared with respective control groups.

**Figure 2:**
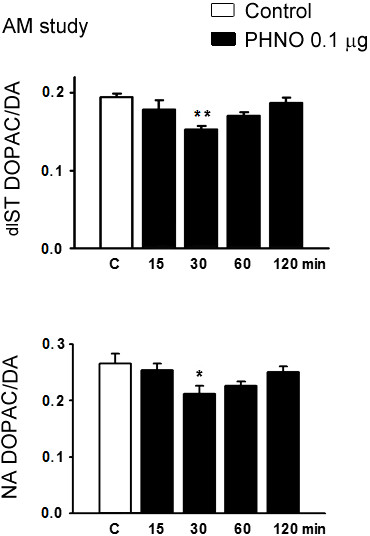
**Effects of PHNO (0.1 μg/3 μl, icv) on dlST (upper) and NA (lower) DOPAC/DA ratios in OVX+E**_**2 **_**rats in the morning.** Data are expressed as mean ± SEM (n = 5–7 rats). *, p < 0.05, **, p < 0.01 compared with respective control groups.

**Figure 3:**
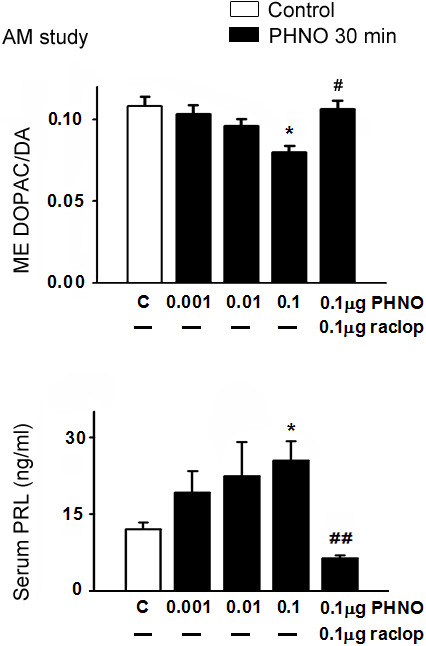
**Effects of PHNO (0.001, 0.01, 0.1 μg/3 μl/rat) and co-administration of PHNO (0.1 μg) with a D**_**2 **_**receptor antagonist, raclopride (0.1 μg), on ME DOPAC/DA ratio (upper) and serum PRL (lower) level in OVX+E**_**2 **_**rats in the morning.** Data are expressed as mean ± SEM (n = 5–7 rats). *, p < 0.05, compared with respective control groups. #, p < 0.05; ##, p < 0.01 compared with the PHNO (0.1 μg)-injected group.

**Figure 4:**
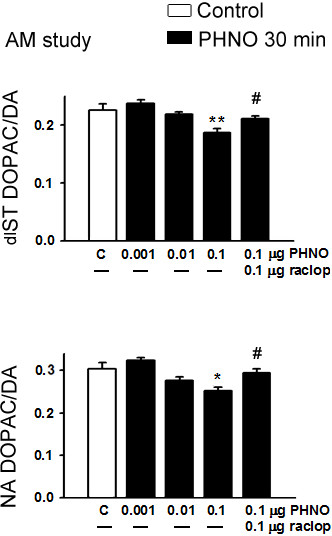
**Effects of PHNO (0.001, 0.01, 0.1 μg/3 μl/rat) and co-administration of PHNO (0.1 μg) with raclopride (0.1 μg) on dlST (upper) and NA (lower) DOPAC/DA ratios in OVX+E**_**2 **_**rats in the morning.** Data are expressed as mean ± SEM (n = 5–7 rats). *, p < 0.05; **, p < 0.01 compared with respective control groups. #, p < 0.05 compared with the PHNO (0.1 μg)-injected groups.

In the time-dependent study, icv injection of 0.1 μg PHNO significantly decreased DOPAC/DA ratios in ME, dlST and NA (p < 0.05 for ME and NA; p < 0.01 for ST; Figures 1 and 2), and increased serum PRL levels (p < 0.05; Figure 1) at 30, but not 15, 60, or 120 min. In the dose–response study, injection of 0.1, but not 0.001 or 0.01 μg PHNO significantly decreased DOPAC/DA ratios in ME, dlST and NA (p < 0.05 for ME and NA; p < 0.01 for dlST; Figures 3 and 4) and increased serum PRL level (p < 0.05; Figure 3) at 30 min. Co-administration with raclopride (0.1 μg) significantly reversed the inhibitory effects of PHNO on DOPAC/DA ratios in ME (p < 0.05; Figure 3), dlST and NA (p < 0.05; Figure 4), and the stimulatory effect of PHNO on serum PRL level (p < 0.01; Figure 3).

### Involvement of D_2_R in diurnal changes of TIDA neuron activity and PRL secretion in OVX+E_2_ rats

**Figure 5:**
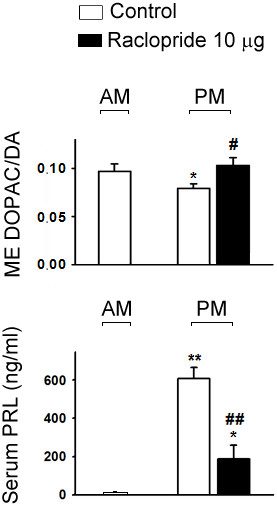
**Effects of a single injection of raclopride (10 μg/3 μl, icv) at 1200 h on ME DOPAC/DA ratio (upper) and serum PRL (lower) level at 1500 h in OVX+E**_**2 **_**rats.** Data are expressed as mean ± SEM (n = 5–7 rats). *, p < 0.05; ** p < 0.01, compared with the control groups in the morning; #, p < 0.05; ##, p < 0.01 compared with respective control groups in the afternoon.

The diurnal changes of TIDA neuron activity and serum PRL level were observed in aCSF-injected rats in which ME DOPAC/DA ratio in the afternoon was significantly lower than that in the morning (p < 0.05; Figure 5) and serum PRL level in the afternoon was significantly higher than that in the morning (p < 0.01; Figure 5). Injection of raclopride (10 μg/3 μl, icv) at 1200 h significantly reversed the lowered ME DOPAC/DA ratio (p < 0.05; Figure 5), and prevented the serum PRL level at 1500 h (p < 0.01; Figure 5). Nevertheless, serum PRL level at 1500 h was still significantly higher than that in the morning (p < 0.05; Figure 5).

### Light- but not E_2_-dependent diurnal expression of D_2_R mRNA in MBH

**Figure 6:**
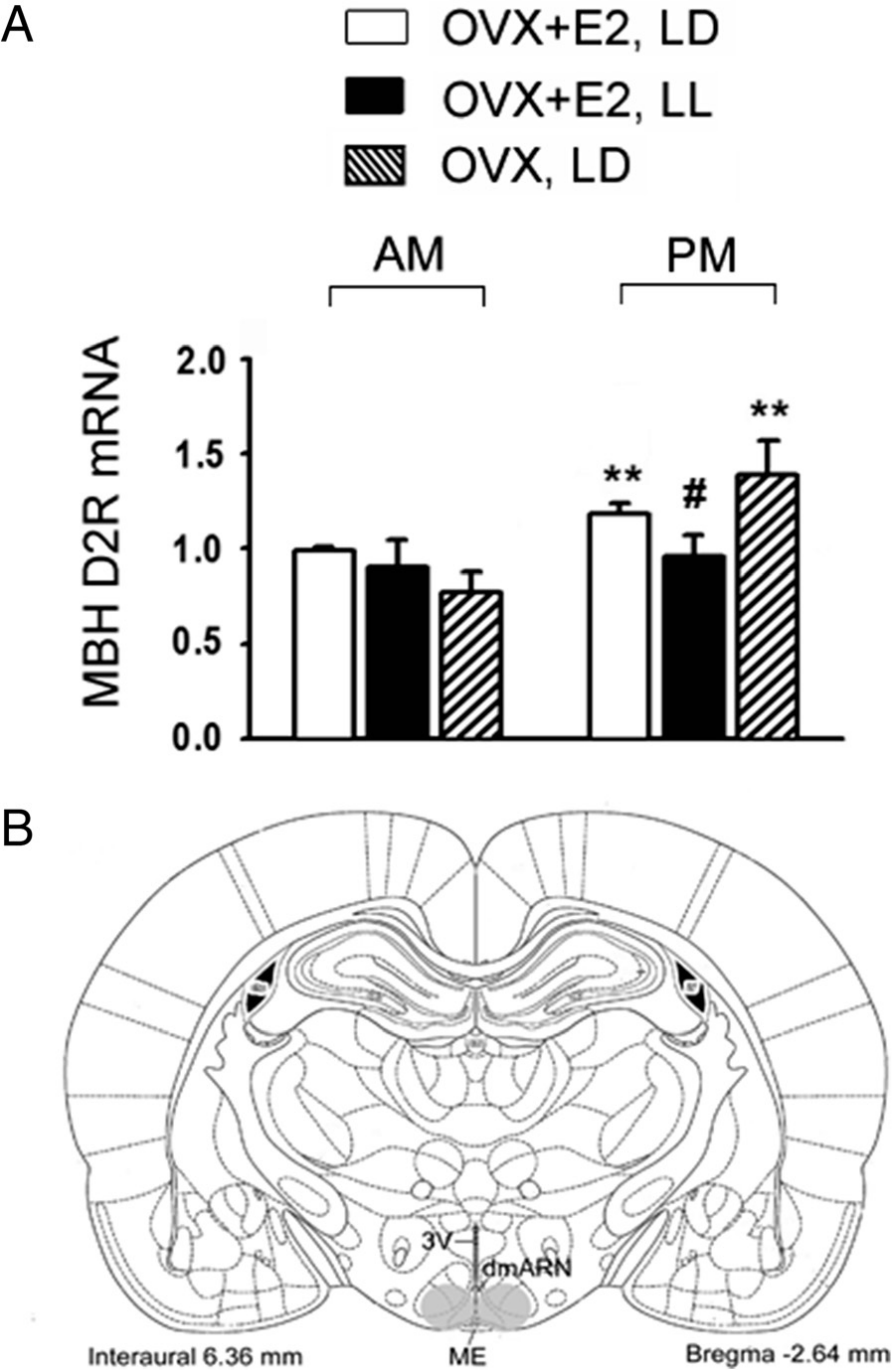
**Morning and afternoon D**_**2**_**R mRNA ratios in MBH and their responses to constant light and E**_**2 **_**exposures (A) and a schematic drawing of the coronal section of the rat brain (B) showing the punched area of MBH [grey circles, [**[15]**]].** A: Data are expressed as mean ± SEM (n = 10–14 rats in each treatment group). **, p < 0.01, compared with the control groups in the morning. #, p < 0.05 compared with the control groups in the afternoon. B: 3 V: the third ventricle; dmARN: dorsal medial arcuate nucleus; ME: median eminence.

The D_2_R mRNA ratio in MBH of OVX+E_2_ rats exhibited a prominent diurnal change, i.e., low in the morning and high in the afternoon (p < 0.01; Figure 6). This rhythm persisted in OVX animals (p < 0.01; Figure 6) but was abolished in OVX+E_2_ animals kept under constant light exposure for 7 days (p < 0.05; Figure 6).

### Effects of AODN and random AODN against D_2_R mRNA on expressions of D_2_R mRNA in MBH, SN and VTA, and on TIDA neuron activities and serum PRL levels in both morning and afternoon of OVX+E_2_ rats

**Figure 7:**
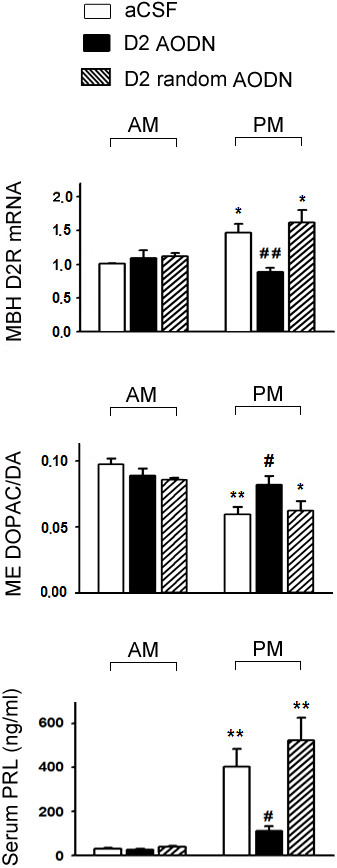
**Effects of antisense oligodeoxynucleotides (AODN) or random AODN (10 μg/3 μl, icv) pretreatment against D**_**2**_**R mRNA for 2 days on MBH D**_**2**_**R mRNA ratios (upper; arbitrary unit), ME DOPAC/DA (middle) and serum PRL (lower) levels both in the morning and afternoon in OVX+E**_**2 **_**rats.** Data are expressed as mean ± SEM (n = 5–7 rats for DOPAC/DA ratio and PRL level; n = 12–14 rats for MBH D_2_R mRNA in each treatment group). *, p < 0.05; ** p < 0.01, compared with respective control groups in the morning; #, p < 0.05; ##, p < 0.01 compared with respective control groups in the afternoon.

The diurnal changes in the expression of MBH D_2_R mRNA (higher in the afternoon), TIDA neuron activity (lower in the afternoon using ME DOPAC/DA as the index) and serum PRL levels (higher in the afternoon) were confirmed in aCSF-treated control rats (Figure 7). Pretreatment with AODN against D_2_R mRNA significantly lowered MBH D_2_R mRNA and serum PRL level (p < 0.01; Figure 7), and increased ME DOPAC/DA ratio (p < 0.05; Figure 7) in the afternoon, while it had no significant effects in the morning. Pretreatment with random AODN against D_2_R mRNA had no significant effect on any of the three variables compared with those in aCSF-treated control (Figure 7).

D_2_R mRNAs in NS and VTA also exhibited a diurnal rhythm with their afternoon levels significantly lower than those in the morning (p < 0.01; Table 1). Pretreatment with AODN significantly lowered morning levels of D_2_R mRNA in NS and VTA (p < 0.05 and 0.01 respectively; Table 1) plus afternoon level in VTA (p < 0.01; Table 1), and disrupted the diurnal rhythm in both regions. Pretreatment with the random AODN had no significant effects on D_2_R mRNA ratios in NS and VTA (Table 1).

**Table 1:** **Dopamine D**_**2 **_**receptor mRNA ratios in SN and VTA of OVX+E**_**2 **_**rats as determined by QRT-PCR**

		**aCSF**	**D**_**2 **_**AODN**	**D**_**2 **_**random AODN**
SN	AM	1.00 ± 0.01	0.76 ± 0.06^#^	1.33 ± 0.13
	PM	0.78 ± 0.02**	0.86 ± 0.03	0.99 ± 0.05*
VTA	AM	1.02 ± 0.02	0.42 ± 0.02^##^	1.38 ± 0.19
	PM	0.77 ± 0.01**	0.46 ± 0.06^#^	0.53 ± 0.08**

Data are expressed as mean ± SEM (n = 8-12 rats in each group). *, p < 0.05; **, p < 0.01, compared with respective control groups in the morning; #, p < 0.05; ##, p < 0.01 compared with respective aCSF-treated groups at the same time point.

Groups of OVX+E_2_ rats received icv injections of aCSF, antisense oligodeoxynucleotides (AODN, 10 μg/3 μl), or random AODN against D_2_ receptor mRNA for 2 days were sacrificed either in the morning or afternoon.

## Discussion

We recently reported that an endogenous DA tone acting on DA D_3_Rs may be involved in diurnal changes of TIDA neuron activity and PRL secretion in OVX+E_2_ rats [10]. We further demonstrated in this study that D_2_Rs were also involved. Moreover, we found that the expression of D_2_R mRNA in the MBH exhibited a diurnal change that persisted in OVX animals but was abolished in OVX+E2 animals kept under constant lighting exposure, so was the response of TIDA neuron activity. To our knowledge, this is the first report showing that a diurnal change in the expression of D_2_R mRNA exists in MBH neurons.

PHNO has been characterized as a highly specific D_2_ receptor agonist which binds primarily to the high-affinity state of D_2_Rs rather than D_3_Rs in the striatum [21] and exhibits a 67-fold selectivity for D_2_Rs versus D_1_Rs [22,23]. The findings that PHNO inhibited the activities of all three central DA neurons tested and its inhibitory effects were blocked by co-administration of raclopride further strengthen the notion that D_2_ autoreceptors are involved in the control of central DA neurons. Raclopride not only blocked the effect of PHNO on TIDA neuron activity, it further lowered serum PRL to below control levels, indicating that not only D_2_Rs but other D_2_R-like receptors may be involved. Due to technical limitations, the time-dependent changes in TIDA neuron activity can only be determined using different groups of rats sacrificed at different time points, but not in individual rats. It is possible that the effect of PHNO was maximal at 30 min and the effects at earlier and later time points may be obscured in group averages.

We used OVX rats with estrogen replacement because the diurnal changes of TIDA neuron activity and afternoon PRL surge are gender specific and induced by estrogen [8]. We have repeatedly shown that the diurnal change in TIDA neuron activity is prerequisite for the estrogen-induced PRL afternoon surge [8,9,24]. Disrupting the TIDA rhythm invariably blunts the PRL surge. The previous [10] and present studies using D_3_R and D_2_R antagonists to disrupt the TIDA rhythm further confirm the notion. It appears that an endogenous DA tone acting on D_2_-like receptors during a critical period of time (from 1200 to 1400 h) is responsible for the lowered TIDA neuron activity in the afternoon.

Although raclopride treatment completely reversed the afternoon decrease of TIDA neuron activity and significantly blunted the PRL surge, it did not block the surge completely, i.e., serum PRL level in the afternoon was still higher than that in the morning. This indicates that factor(s) other than withdrawal of DA inhibition stimulates the PRL secretion. It is well-established that decreased inhibition by DA (PRL-inhibiting hormone) plus increased stimulation by a putative PRL-releasing hormone are required for a full-blown PRL surge [25-27]. The present finding further substantiates the notion.

That the mRNA of D_2_Rs in MBH exhibited a diurnal rhythm and was disrupted by constant light exposure are novel findings. It appears that when TIDA neuron activity was high, the expression of D_2_R mRNA was low, and vice versa. Moreover, the findings that both D_2_R mRNA and TIDA diurnal rhythms are sensitive to constant light exposure but persist in OVX rats are consistent with the criteria of true circadian rhythms [8,28]. Pretreatment with an AODN against D_2_ receptor mRNA not only reversed the increased expression of D_2_ receptor mRNA in the afternoon, but also prevented the diurnal changes of TIDA neuron activity and PRL secretion. This finding indicates that the diurnal change in the expression of D_2_R mRNA may underlie the changes of TIDA neuron activity and in turn the PRL surge, although the exact mechanism needs further work.

This study raised another interesting issue on the origin of dopamine acting on TIDA neurons. Dopamine may arise from recurrent collaterals of TIDA neurons or from other hypothalamic DA neurons including periventricular-hypophysial (PH) and tuberohypophysial (TH) DA neurons [29]. It has been shown that rhythm-related genes are present not only in suprachiasmatic nucleus (SCN) and ARN [30], but also in hypothalamic DA neurons, including TIDA, PHDA and THDA neurons [31], indicating that these neurons may generate their own rhythms. Single-unit recording of DA-responsive dmARN neurons *in vitro* also reveals a diurnal rhythm of their firing rates [7]. On the other hand, it is well-established that SCN is the master biological clock that coordinates most circadian rhythms observed in mammals and bilateral lesions of the SCN eliminate both TIDA diurnal rhythm and the estrogen-induced PRL surge [9], indicating an entraining signal originated from SCN can affect directly or indirectly on TIDA neurons.

Circadian changes of midbrain DA neurons’ activities in rats have been reported with their levels higher during dark hours when the animals exhibit nocturnal motor activities [28,32]. Recent studies have shown that striatal D_2_/D_3_R proteins and D_3_R mRNA levels in mouse also exhibit diurnal rhythms [11,12]. Our findings that D_2_R mRNAs in SN and VTA exhibited a morning high, afternoon low rhythm are in agreement with the changes of D_3_R mRNA levels in mouse striatum [12]. That the D_2_R mRNAs in SN and VTA were significantly lowered by pretreatment of AODN against D_2_R mRNA further confirms the knockdown effects of AODN.

## Conclusions

The present study demonstrates that D_2_Rs are involved in the inhibitory actions of exogenous and endogenous dopamine on TIDA neuron activity. Moreover, a diurnal change of D_2_R mRNA expression in the MBH may be involved in the diurnal rhythms of TIDA neuron activity and PRL secretion in OVX+E_2_ rats.

## Competing interests

The authors declare that they have no competing interests.

## Authors’ contributions

SLL designed and carried out all studies except QRT-PCR, and analyzed, plotted all the data and drafted the manuscript. SCH carried out QRT-PCR study and participated in its design. JTP conceived of the study, participated in its design and coordination, and revised the manuscript. All authors read and approved the final manuscript.
